# Optimizing the supply of vaccines for COVID-19

**DOI:** 10.2471/BLT.21.287513

**Published:** 2021-12-01

**Authors:** Angela K Shen, Ann Lindstrand, Benjamin Schreiber, Kate O’Brien

**Affiliations:** a Health Emergencies Programme, World Health Organization, Avenue Appia 20, 1211 Geneva 27, Switzerland.; b Department of Immunization, Vaccines and Biologicals, World Health Organization, Geneva, Switzerland.; c Programme Division, United Nations Children’s Fund, New York, United States of America.

The global health community and the COVID-19 Vaccine Global Access (COVAX) initiative – the partnership co-led by Gavi, the Vaccine Alliance, the Coalition for Epidemic Preparedness Innovations, the World Health Organization (WHO) and the United Nations’ Children’s Fund (UNICEF) – have focused on securing vaccines for the coronavirus disease 2019 (COVID-19) to equitably vaccinate the world’s population. Besides the complex task of securing and distributing billions of doses for vaccination programmes, country readiness to administer these doses is a daunting challenge. Vaccination requires more than just vaccines. 

UNICEF, WHO and Gavi have established a COVID-19 vaccination Country Readiness and Delivery^1^ workstream to support country readiness for COVID-19 vaccination programmes as part of COVAX. 

To optimize the use of COVID-19 vaccines, the country readiness and delivery work has focused on developing tools, guidance and processes to support countries to receive and deliver COVID-19 vaccines. The aim of these efforts is to achieve equitable global access to COVID-19 vaccines as the global roll-out of COVID-19 vaccines is the fastest and largest deployment of vaccines in history. Achieving the aim of vaccinating 70% of the world’s population by June 2022 would be an extraordinary logistical feat.^2^ As of 4 November 2021, 7.15 billion doses of COVID-19 vaccines have been administered. However, these doses have been unequally distributed and the world is far from reaching global goals.^3^ Many vaccination programmes have been constrained by supply, but this situation is about to change as projections expect supply to increase exponentially in the last months of 2021 and in 2022.^2^^,^^4^

Preparing countries (regardless of who is financing the vaccine doses) is key to the success of vaccination programmes. Developing vaccine campaigns can take years but has now been contracted to months.^5^^,^^6^ As more vaccine doses flow into countries, implementation challenges are emerging, and addressing such challenges at the operational level is important. For most countries, adults are a new target population for vaccination and the design of appropriate strategies for this group is required. In addition, some vaccine products are complex in storage and handling. Systems to monitor products and processes, and identify and vaccinate people, may be weak in some countries, adding complexity to existing challenges, as many health systems are struggling to maintain essential health services. Last, governments need to provide training and supervision of health-care workers, support demand generation and communicate about vaccines. 

The key to mitigating and eventually ending the pandemic is reaching high levels of vaccination coverage as quickly as possible. The country readiness and delivery workstream has the critical role of supporting the identification of implementation bottlenecks and coordinating a strategic response to address challenges. Building lessons learnt from efforts in countries who received initial doses, the workstream seeks to achieve its mission through two functions. First, through the provision of technical guidance and provision of resources and tools such as communication toolkits, budget templates, readiness checklists and training videos on how to administer the different vaccines. This support will enable strategic coordination and implementation at the country level, together with technical implementing partners, to vaccinate their populations.^7^ Through its convening role, the members of the workstream engage technical partners to address gaps in countries, building on the backbone of routine immunization programmes, as countries and partners have long-standing expertise in introducing new vaccines.^5^ Second, through extensive collaboration with donors to secure operational funding to countries for roll-out of doses in a clear and transparent way. The workstream convenes stakeholders, many of whom raise funds to support vaccine purchase and delivery, with the aim of ensuring sufficient funds for countries to vaccinate their populations.

To optimize implementation efforts at country level, four sub-workstreams function under the country readiness and delivery structure in addition to the other technical workstreams on data, demand, supply chain and logistics, and innovation. These four sub-workstreams are: (i) the implementation monitoring review, which seeks to enhance country-level intelligence and progress monitoring; (ii) country support teams, who build on weekly insights on implementation activities to focus additional operational support for vulnerable countries; (iii) a bi-weekly funders forum where donors seek to address implementation bottlenecks through coordinated funding; and (iv) a newly created Implementers Action Network, a mechanism to increase operational coordination across key implementing partners in countries.

While these workstreams and sub-workstreams all strive to coordinate and leverage global resources towards country support, countries ultimately decide how to vaccinate their populations. However, it is a global responsibility to support the collective good of the world and to ensure countries have the tools and means to vaccinate. To succeed in mitigating the pandemic, the health and development community must be coordinated to successfully support countries effectively. 
